# Continuous glucose monitoring empowers adolescents to take responsibility of diabetes management

**DOI:** 10.4102/phcfm.v15i1.3879

**Published:** 2023-04-12

**Authors:** Letitia Williams, Elmarí Deacon, Esmé Van Rensburg, David Segal

**Affiliations:** 1 Compres Research Focus Area, Faculty of Health Science, North-West University, Potchefstroom, South Africa; 2 Optentia Research Unit, Faculty of Health Science, North-West University, Potchefstroom, South Africa

**Keywords:** adolescents, continuous glucose monitoring (CGM), empowerment, illness perception, diabetes management

## Abstract

**Background:**

Managing diabetes is especially challenging for adolescents, and they often struggle to believe they can manage the condition. Illness perception has been widely associated with better diabetes management outcomes, but the influence of continuous glucose monitoring (CGM) on adolescents has been largely neglected.

**Aim:**

The study aimed to explore the illness perception of a group of adolescents living with type 1 diabetes (T1D) using CGM.

**Setting:**

The study was conducted at a medical centre that provides diabetes care services to youth living with T1D in Parktown, South Africa.

**Methods:**

A qualitative research approach using semi-structured online interviews was used to gather data that was thematically analysed.

**Results:**

Themes emerging from the data confirmed that CGM creates a sense of control over diabetes management as blood glucose measures were more visible. A sense of normalcy was established as CGM influences a new routine and a way of life, integrating diabetes into a young person’s identity. Despite the users’ awareness of being different due to diabetes management, CGM assisted in creating a sense of belonging, contributing to developing a better quality of life.

**Conclusion:**

Findings of this study support the use of CGM as a means of empowering adolescents struggling with diabetes management to achieve better treatment outcomes. The important role of illness perception in facilitating this change was also evident.

**Contribution:**

By listening to the adolescent’s voice, CGM was identified as a possible intervention to empower adolescents to improve diabetes management.

## Introduction

Illness perception has been widely acknowledged as integral to self-care management behaviours and adherence to diabetes management^1,2,3,4^ and refers to an acquired set of beliefs regarding one’s illness obtained by gathering information and individual interpretations of personal experiences of living with a disease or illness.^2,5^ The best-known model of illness perception, the Common-Sense Model (CSM), is regarded as a parallel processing model, which posits that individuals develop perceptions of illness regarding their diagnosis and management requirements through cognitive and emotional representations that inform the coping strategies employed and influence future outcomes.^6,7^ The CSM proposes five cognitive dimensions, which guide the formation of illness representations and subsequent outcomes, namely: (1) identity, (2) perceived cause, (3) consequences, (4) control and/or cure and (5) timeline.

Adolescence, in the context of this study, is seen as a transition phase where the individual is expected to take more responsibility for their diabetes care plan^8^ than during previous developmental stages. It is also known as a stage with high rates of non-adherence and relatively poor health outcomes,^9^ which necessitates an understanding of modifiable factors to address such consequences.^3^ One of these factors could be illness perception. In two related studies, the illness perception of adolescents with well-controlled and uncontrolled diabetes was investigated. Jonker^10^ reports that adolescents with well-controlled diabetes viewed the condition as manageable and a part of their lives. In contrast, Lesage^11^ reports that adolescents with uncontrolled diabetes viewed the condition’s management as challenging and were mostly motivated by fear to adhere to the diabetes care plan. It is also important to note that in both studies participants reported that managing diabetes made them ‘different’ from their peers, adding to the experience of emotional distress.^10,11^

Technological advancements in blood glucose monitoring have led to the development of continuous glucose monitoring (CGM), which provides a sustained, visible measure of variations of the individual’s glucose levels in real time.^12,13^ Research has shown possible treatment outcomes improvement when using CGM,^14,15^ while the psychological barriers and benefits that accompany the use of CGM systems have indicated that employing this facility to form part of the diabetes care plan holds multiple benefits for the users.^16^ For example, patients using CGM have an increased chance of achieving greater control over managing their blood glucose levels, which, in turn, offers the possibility of decreased levels of fear, improved quality of life and a sense of empowerment.^16,17^

The literature focuses on the benefits of CGM in paediatric patients and caregivers,^18,19,20^ with limited reference to the use of CGM in adolescents or the influence of the monitoring facility on illness perception in this developmental phase where illness perception is formed.^21,22^ The study aimed to explore the illness perception of a group of adolescents living with type 1 diabetes (T1D) using CGM.

## Research methods and design

### Study design

A descriptive, exploratory qualitative research design within a social constructivist approach was used to explore the illness perception of adolescents using CGM. This approach allowed for a more in-depth study of the experiences of the adolescents, while acknowledging how meaning is created through the use of language in the social contexts in which adolescents function.^23^

### Setting, population and sampling

The target population included adolescents living with T1D using CGM. Recruitment was carried out at the Centre for Diabetes and Endocrinology (CDE) in Parktown, South Africa, which specialises in paediatric diabetes. Non-random purposive sampling was utilised to select participants that adhered to the following criteria: (1) adolescents aged between 12 and 17 years old; (2) diagnosed with T1D for more than 12 months preceding data collection and (3) who were patients at the CDE: Parktown, Johannesburg and (4) have been using CGM for more than 3 months. Furthermore, they needed access to an internet connection and be willing to participate in the online semi-structured interviews in English or Afrikaans. Adolescents undergoing psychotherapy at the time of the study were ineligible as the psychotherapeutic process might have had an impact on their perceptions of diabetes management. Adolescents living with another chronic medical condition were also ineligible as their lived experience of managing it might have affected their lived experience of managing T1D.

Prospective participants were invited to take part in the study by means of an information leaflet distributed by the CDE. Those interested indicated that via e-mail, after which a screening process followed. In total, seven participants indicated a willingness to participate. None of the eligible participants declined to take part in the study. No participants dropped out after the screening.

### Data collection and interview guide

Data were collected from October to November 2020. Seven semi-structured interviews were conducted. As the coronavirus disease 2019 (COVID-19) pandemic limited face-to-face interaction, interviews were held online to ensure the safety of participants. All data gathering took place in the participant’s preferred secure and confidential setting. Interviews lasted 30 min–40 min and were recorded on an online video conferencing platform. No parents chose to sit in the interviews. The interviews were guided by the following prompts: (1) Tell me more about yourself? (age, time since diagnosis, time using CGM and how you currently manage diabetes); (2) Tell me about your experience of living with diabetes? (which aspects of your life are affected by T1D); and (3) Tell me about your experience of living with diabetes while using CGM? (what makes the use of CGM easier and/or more difficult?; what are the advantages and disadvantages of using CGM?; how did using GGM change how you view living with diabetes?). The first author conducted all the interviews, had no relationships with the participants and was trained to conduct the interviews. The participants also did not know each other. The first author had limited expert knowledge on diabetes management but regularly consulted with experts in the process of writing-up of results.

Data were transcribed and analysed after each interview. Data saturation was reached after the fifth interview when no new themes emerged. The last two interviews were conducted to confirm the themes that had emerged.

### Data analysis

Data were transcribed and analysed using thematic analysis at the outset of the interview process.^24,25^ The first author transcribed the data as part of immersion into the data gathered. The transcripts were checked by the second author, who also assisted with the analysis. No computer software was used in the process. The analysis process involved: (1) familiarisation of the data, (2) initial code generation, (3) theme searching, (4) theme reviewing, (5) defining and naming themes and (6) report writing.

### Trustworthiness

The first author kept reflective notes on her experiences during interviews.^26^ Credibility was achieved by using the most appropriate methods for data collection, peer discussions amongst authors and the inclusion of adequate participant quotations to support the findings. Confirmability was enhanced by the first author reflecting on her limitations and biases, as well as triangulation, referring to the collection of data from observations, recordings, interviews and literature to create a deeper understanding of the themes. The second author was involved in the co-coding of the data and interpretation of data under codes, which assisted in ensuring dependability. This was further enhanced by ample field notes made during the interviews. Transferability was achieved by providing contextual information and gaining in-depth explanations from the participants to form a better understanding of their experiences.

### Ethical considerations

Ethical approval was obtained from the Health Research Ethics Committee (HREC) (NWU-00125-18-A1) of the North-West University, South Africa. As the participants were under the age of 18 years and had been diagnosed with diabetes, the study was considered medium risk. Participants and their caregivers were fully informed of the voluntary nature of the study, and the anonymity and confidentiality of their identities were guaranteed. Delayed written informed consent was obtained from the individuals and/or minors’ legal guardian or next of kin before interviews commenced. This was necessitated by lockdown restrictions during the COVID-19 pandemic and entailed that the parent and adolescent both signed the consent form while engaged in the online interview with the researcher, with all three parties having a witness present to co-sign. The participants then scanned or faxed the consent forms or a photo of the form, which was sent to the independent person obtaining the consent. Interviews were conducted online, which allowed participants to arrange the interview at a time and space that is comfortable for them. Participants were informed of their rights and reminded of the voluntary nature of their participation. Anonymity was assured by storing the consent forms separately from the participant code list.

## Results

Table 1 provides a summary of the characteristics of the participants in the study. The study population consisted of three males and four females, between the ages of 14 and 16 years (mean age 14.9), two Afrikaans speakers and five English speakers. The mean age at which they were diagnosed with T1D was 7 years (mean duration living with diabetes was 7 years) with the mean duration of CGM use 3.4 years. Three themes emerged from the data: (1) CGM creates a sense of control over diabetes management; (2) CGM assists in incorporating diabetes management into their identity and (3) CGM created opportunities for positive outcomes.

**TABLE 1:** Characteristics of participants.

Pseudonym	Sex	Age	Time since diagnosis (years)	Time using CGM (years)
Amelia	Female	14	2	2
Ava	Female	14	4	4
Darla	Female	15	14	2
Hanna	Female	15	10	3
Harry	Male	15	10	5
Ian	Male	15	4	4
Ryan	Male	16	5	4

CGM, continuous glucose monitoring.

### Theme 1: Continuous glucose monitoring creates a sense of control over diabetes management

Participants reported that the use of CGM promoted a sense of control over their diabetes management. This sense of control was driven by the evidence of blood glucose levels provided by CGM. Being able to ‘see’ sensor glucose levels, and predict what levels will be in the future, not only assisted in their normal daily activities but also reduced the likelihood of medical emergencies. Experiencing a sense of control empowered the participants to take more responsibility for their diabetes management.

Control was facilitated by the visibility; and ‘being aware of [*their*] sugar 24/7’ (Hanna, age 15, female) in real time helps them to predict the direction of their blood glucose levels without interfering with their activities, ‘because now [*they*] can constantly see [*their*] levels without having to stop what [*they are*] doing’ (Darla, age 15, female). Predictability and ‘knowledge of where [*their blood glucose levels*] are at that time’ (Amelia, age 14, female) also assisted in their anticipating possible medical emergencies as ‘it helps you a lot to look at your sugar so that it doesn’t go too high or low’ (Harry, age 15, male). As participants were aware of the direction of their glucose levels pertaining to CGM’s predictions of ‘where it’s going, where it might be, if [*they are*] high or low’ (Amelia, age 14, female) it allowed them to intervene in time anticipating possible medical emergencies.

The experience of more control over diabetes management led to most participants becoming ‘a lot more responsible’ and disciplined. They matured and embraced the hands-on experience of managing the CGM, as one participant noted: ‘[*CGM readings*] sort of puts you in the mindset of wanting to control it better’ (Hanna, age 15, female). Darla, a 15 year old female, said: ‘Since I’ve been using the CGM, it’s life-changing’, which promoted overall improved diabetes management, illness perceptions and healthy lifestyles. This sense of control over their diabetes management decreased their ‘worry about high or low blood sugar’ (Harry, age 15, male) and promoted a sense of self-confidence in their abilities because they felt empowered and ‘more motivated to succeed’ (Ian, age 15, male). As Amelia, a 14 year old female commented: ‘I’ve realised that I’m the boss’. All study subjects noted that they ‘see a very big difference’ (Hanna, age 15, female) because ‘[*they*] feel like they have a little bit more control over it. So they can calm down’ (Ava, age 14, female) as their ‘[*blood glucose levels are*] even more controlled now’ (Darla, age 15, female).

### Theme 2: Continuous glucose monitoring assists in incorporating diabetes management into their identity

All participants reported that the use of CGM made living with and managing T1D more convenient. They could incorporate the management of diabetes and using CGM into their routines and create a new ‘normal’ way of life, integrating diabetes into their identities.

All the adolescents noted that CGM was easy to use and incorporate into their daily routine:

‘It’s become part of everyday life and I don’t even notice it. It’s like routine to me and it’s like something normal, it’s like brushing your teeth to me.’ (Darla, age 15, female)

which:

‘just made [*diabetes management*] a lot more convenient and easier.’ (Hanna, age 15, female)

They also reported:

‘[*T*]hat now with the CGM […] it’s like not so many disruptions in my life’, which allowed them to take part in more activities than they used to and ‘makes [*them*] feel that [*they*] can live close to a normal life’ (Hanna, age 15, female)

by engaging in the activities of an average teenager.

The sense of control created by the use of CGM led to adolescents taking part in the study being able to integrate the management requirements of their diabetes into their identity, which resulted in a new ‘normal’ and ‘made it seem less like a disability’ (Hanna, age 15, female). This had a role to play in them coming to terms with and accepting their diagnosis and its management, which was reflected in Amelia’s and Darla’s responses:

‘I have properly adjusted to my new life.’ (Amelia, age 14, female)

‘this is my whole life […]. I can either be sad and sit in my room and cry about it all the time […] or I should be happy and carry on with my life as I’m going.’ (Darla, age 15, female)

The use of CGM was seen as being an integral ‘part of [*themselves*]’ (Ryan and Amelia) and their way of life, and not just as mere habit or routine.

### Theme 3: Continuous glucose monitoring creates opportunities for positive outcomes

Continuous glucose monitoring is a visible device on the body and wearing it attracts attention. All participants agreed that diabetes management made them ‘different’ from other adolescents, but it also creates opportunities for educating others and relating to others using CGM. Six adolescents in this study noted that they had been somewhat self-conscious about the use and visibility of the CGM device at first, as explained by Darla:

‘I’ve got like a tan […] where the CGM was. And it looks a bit funny and that I’m a bit self-conscious about.’ (Darla, age 15, female)

But as time passed, their perspective changed, as Amelia, noted: ‘It doesn’t bother me at all’. Most participants did, however, say that at times they still experienced ‘a little bit of [*self-consciousness*] about [*CGM’s visibility*] but not to the extent that it was [*previously*]’ (Hanna, age 15, female), and they would try to ‘hide it in a way’ (Darla, age 15, female). They capitalised on this difference and noted that when the CGM was visible others would ‘ask [*them*] what’s that on your arm? What does it do? […] [*they*] always tell them […] what it is and what it does’ (Amelia, age 14, female) and ‘[*they*] are really happy to share it’ (Darla, age 15, female). This awareness-raising, in turn, ‘brings more self-confidence. Because it’s nice to educate people sometimes’ (Hanna, age 15, female).

Participants also noted that they ‘get excited’ (Amelia, age 14, female) and experience a sense of belonging and community when they saw someone else wearing a CGM. They were able to connect with other individuals who shared similar experiences, both in person, for example, ‘at school’ (Amelia, age 14, female and Hanna, age 15, female) or at ‘diabetes camp’ (Ryan, age 16, male) and on social media platforms (Amelia, age 14, female; Ava, age 14, female; Ryan, 16 year old male). Amelia shared:

‘I follow a lot of diabetes pages on Instagram as well. So I get daily tips and tricks. Stories from other people as well. So it’s awesome. Cause you’ve got a community of people who understand what you are going through as a person. And it’s great to hear about other people’s experiences as well.’ (Amelia, age 14, female)

## Discussion

What we learned from the adolescents is that using CGM could potentially empowered them to manage their diabetes and incorporate diabetes management into their daily lives. Although the study followed an inductive approach, the following dimensions of the CSM were prominent in the results: cognitive representations of identity, control and/or cure as well as emotional representations.

Representation of identity refers to an individual’s illness concepts, understanding of the illness and its management and how meaning is formed and ascribed.^22^ In this study, participants were able to incorporate both the diagnosis and the diabetes care plans with using CGM as a way of life, by regarding it as normal and an integral part of themselves. This is similar to findings from Jonker,^10^ who found that a group of adolescents living with well-controlled diabetes reported that T1D becomes a way of life. The literature suggests that this normalisation and eventual acceptance of their diabetes care plans may further reduce their experiences of being different, promote better treatment adherence, and ultimately improve their overall quality of life.^27^

This new normal was created over time and involved an emotional progression from a range of negative emotions (such as disappointment, sadness and frustration due to feeling different from their peers) to experiencing a sense of normalcy in their daily lives since using CGM, specifically because medical emergencies were less likely with proper management. Negative emotions experienced by adolescents living with T1D are well documented,^28,10,11^ but it is important to note that participants in our study also reported feeling empowered by using the monitoring device and experiencing less anxiety as CGM allows individuals to better manage and intervene in their diabetes care plans as it results in the achievement of predictability and control.^16^ This feeling of empowerment is similar to the findings of Jonker,^10^ who reported that adolescents living with well-controlled diabetes perceive T1D to be manageable and within their control.

The control domain pertaining to the beliefs regarding the illness’s controllability was prominent in this study.^22^ These perceptions prompt individuals to adopt specific disease-modifying and management behaviours for better treatment outcomes and to comply with the diabetes care plans.^29,30^ In our study, a sense of control and predictability of blood glucose levels resulted from using CGM, motivating improved treatment adherence. This is in stark contrast with the study of adolescents living with uncontrolled T1D being motivated to adhere to diabetes care plans by fear.^11^ The difference in motivating factors for treatment adherence is crucial for the adolescents’ well-being – being motivated by fear leads to negative psychological outcomes, whereas our group experienced being empowered by CGM to manage their diabetes better. This finding is similar to those of authors who reported that as adolescents felt more in control of diabetes management, they were encouraged to accept more responsibility for their diabetes management.^31,32^ Our study, however, adds that this confidence can be facilitated by the use of CGM, which has not been reported in people at this developmental stage before. Also, adolescents in our study were able to change the feeling of being different (as a result of wearing CGM) into an opportunity to create awareness and a sense of belonging to those living with T1D. Often, living with T1D is a private experience with adolescents preferring that others do not know of their condition, whereas wearing a visible CGM device created opportunities for positive interaction and empowerment in the study participants.

A major limitation in the study is the small sample size and lack of diversity of participants. Having a larger sample size and more diverse demographic variability is needed to understand the experiences of different socio-demographic and cultural groups. The fact that the participants were using CGM for an extended period could be an indication of their belonging to a higher socioeconomic group, which should be acknowledged in the study. Future studies should focus on including socioeconomic groups that are representative of South Africa. The value in what we report is in the increased understanding of the role illness perception plays in adolescents living with T1D, using CGM. The role of visibility and predictability of blood glucose should be emphasised by diabetes educators to highlight the benefits of using this monitoring device and facilitate awareness of ways that using CGM can change the way an adolescent thinks about diabetes management. The experiences of participants who struggled to adjust to using CGM should be investigated as a follow-up study because the use of this technology can offer very obvious benefits. Also, investigations of the cost and availability of CGM should be conducted to determine the feasibility of using CGM in adolescents more widely as the technology can be expensive to install and maintain, depending on the financial support for the medical services available to users.

## Conclusion

This study demonstrated that adolescents using CGM experienced a sense of control over diabetes, which resulted in them feeling empowered to manage the condition and to incorporate it into their identities. Adolescents living with T1D are particularly at risk of poor adjustment and adherence to diabetes management as they struggle to incorporate living with the condition into their identities. This study, therefore, proposes that using CGM could assist with identity development and mastery of living with diabetes through achieving control of blood glucose. It further confirms that positive psychological outcomes, such as feeling empowered, are possible for adolescents living with T1D, despite being different and having to adhere to diabetes care plans.

## References

[1] Broadbent E, Donkin L, Stroh JC. Illness and treatment perceptions are associated with adherence to medications, diet, and exercise in diabetic patients. Diabetes Care. 2011;34(2):338–40. 10.2337/dc10-1779 21270191 PMC3024345

[2] Hagger V, Trawley S, Hendrieckx C, et al. Diabetes MILES Youth-Australia: Methods and sample characteristics of a national survey of the psychological aspects of living with type 1 diabetes in Australian youth and their parents. BMC Psychol. 2016;4(1):42. 10.1186/s40359-016-0149-9 27519408 PMC4983064

[3] McGrady ME, Peugh JL, Hood KK. Illness representations predict adherence in adolescents and young adults with type 1 diabetes. Psychol Health. 2014;29(9):985–98. 10.1080/08870446.2014.899361 24588345

[4] Mc Sharry J, Moss-Morris R, Kendrick T. Illness perceptions and glycaemic control in diabetes: A systematic review with meta-analysis. Diabet Med. 2011;28(11):1300–10. 10.1111/j.1464-5491.2011.03298.x 21418098

[5] Petrie KJ, Weinman J. Patients’ perceptions of their illness: The dynamo of volition in health care. Curr Dir Psychol Sci. 2012;21(1):60–5. 10.1177/0963721411429456

[6] Broadbent E, Petrie KJ, Main J, Weinman J. The brief illness perception questionnaire. J Psychosom Res. 2006;60(6):631–7. 10.1016/j.jpsychores.2005.10.020 16731240

[7] Moss-Morris R, Weinman J, Petrie K, Horne R, Cameron L, Buick D. The revised illness perception questionnaire(IPQ-R). Psychol Health. 2002;17(1):1–16. 10.1080/08870440290001494

[8] Gonzalez JS, Tanenbaum ML, Commissariat PV. Psychosocial factors in medication adherence and diabetes self-management: Implications for research and practice. Am Psychol. 2016;71(7):539–51. 10.1037/a0040388 27690483 PMC5792162

[9] Hilliard ME, Wu YP, Rausch J, Dolan LM, Hood KK. Predictors of deteriorations in diabetes management and control in adolescents with type 1 diabetes. J Adolesc Health. 2013;52(1):28–34. 10.1016/j.jadohealth.2012.05.009 23260831 PMC4467546

[10] Jonker D, Deacon E, Van Rensburg E, Segal D. Illness perception of adolescents with well-controlled type 1 diabetes mellitus. Health Psychol Open. 2018;5(2):2055102918799968. 10.1177/2055102918799968 30245842 PMC6146331

[11] Lesage SS, Deacon E, Van Rensburg E, Segal D. ‘It kinda sucks’: Illness perception of a group of South African adolescents with type 1 diabetes mellitus. Afr J Prim Health Care Fam Med. 2021;13(1):e1–e9. 10.4102/phcfm.v13i1.2782 PMC8007989 33764139

[12] Bode BW, Battelino T. Continuous glucose monitoring in 2016. Diabetes Technol Ther. 2017;19(S1):S11–S18. 10.1089/dia.2017.2502 28192020

[13] Reiterer F, Polterauer P, Schoemaker M, et al. Significance and reliability of MARD for the accuracy of CGM systems. J Diabetes Sci Technol. 2017;11(1):59–67. 10.1177/1932296816662047 27566735 PMC5375072

[14] Laffel LM, Kanapka LG, Beck RW, et al. Effect of continuous glucose monitoring on glycemic control in adolescents and young adults with type 1 diabetes: A randomized clinical trial. J Am Med Assoc. 2020;323(23), 2388–96. 10.1001/jama.2020.6940 PMC7298603 32543683

[15] Agarwal S, Cappola AR. Continuous glucose monitoring in adolescent, young adult, and older patients with type 1 diabetes. J Am Med Assoc. 2020;323(23):2384–85. 10.1001/jama.2020.7058 PMC7385810 32543670

[16] Halford J, Harris C. Determining clinical and psychological benefits and barriers with continuous glucose monitoring therapy. Diabetes Technol Ther. 2010;12(3):201–5. 10.1089/dia.2009.0121 20151770

[17] Litchmann ML, Allen NA. Real-time continuous glucose monitoring facilitates feelings of safety in older adults with type 1 diabetes: A qualitative study. J Diabetes Sci Technol. 2017;11(5):988–95. 10.1177/1932296817702657 28376647 PMC5950993

[18] Boyce E. Knowledge is power? How continuous blood glucose monitoring systems are changing the management of Type 1 diabetes mellitus. Pediatr Nurs. 2020;46(4):179–95.

[19] Franceschi R, Micheli F, Mozzillo E, et al. Intermittently scanned and continuous glucose monitor systems: A systematic review on psychological outcomes in pediatric patients. Front Pediatr. 2021;9:660173. 10.3389/fped.2021.660173 34026692 PMC8131655

[20] Rusak E, Ogarek N, Wolicka K, et al. The Quality of Life and satisfaction with continuous glucose monitoring therapy in children under 7 years of age with T1D using the rtCGM system integrated with insulin Pump-A caregivers point of view. Sensors (Basel). 2021;21(11):3683. 10.3390/s21113683 34070638 PMC8198889

[21] Lawton J, Blackburn M, Allen J, et al. Patients’ and caregivers’ experiences of using continuous glucose monitoring to support diabetes self-management: Qualitative study. BMC Endocr Disord. 2018;18(1):12. 10.1186/s12902-018-0239-1 29458348 PMC5819241

[22] Leventhal H, Benyamini Y, Brownlee S, et al. Illness perceptions: Theoretical foundations. In: Petrie KJ, Weinman J, editors. Perceptions of health and illness: Current research and applications. Amsterdam: Harwood Academic, 1997; p. 19–45.

[23] Losantos M, Montoyau T, Exeni S, Loots G & Santa Cruz M. Applying social contructionist epistemology to research in psychology. Int J Collab Pract. 2016;6(1):29–42.

[24] Braun V, Clarke V. Using thematic analysis in psychology. Qual Res Psychol. 2006;3(2):77–101. 10.1191/1478088706qp063oa

[25] Braun V, Clarke V. Teaching thematic analysis: Overcoming challenges and developing strategies for effective learning. Psychologist [serial on the Internet]. 2013 [cited 2021 Aug 16];26(2), 120–3. Available from: https://uwe-repository.worktribe.com/output/937596.

[26] Lincoln YS, Guba EG. Paradigmatic controversies, contradictions, and emerging confluences. In: Denzin NK, Lincoln YS, editors. The handbook of qualitative research, 2000; 2nd ed., pp. 1065–122), Thousand Oaks, CA: Sage.

[27] Commissariat PV, Kenowitz JR, Trast J, Heptulla RA, Gonzalez JS. Developing a personal and social identity with type 1 diabetes during adolescence: A hypothesis generative study. Qual Health Res. 2016;26(5):672–84. 10.1177/1049732316628835 26893304 PMC9639364

[28] Buchberger B, Huppertz H, Krabbe L, Lux B, Mattivi JT, Siafarikas A. Symptoms of depression and anxiety in youth with type 1 diabetes: A systematic review and meta-analysis. Psychoneuroendocrinology. 2016;70:70–84. 10.1016/j.psyneuen.2016.04.019 27179232

[29] Fortenberry KT, Berg CA, King PS, et al. Longitudinal trajectories of illness perceptions among adolescents with type 1 diabetes. J Pediatr Psychol. 2014;39(7):687–96. 10.1093/jpepsy/jsu043 24934247 PMC4161993

[30] McAndrew LM, Musumeci-Szabó TJ, Mora PA, et al. Using the common sense model to design interventions for the prevention and management of chronic illness threats: From description to process. Br J Health Psychol. 2008;13(Pt 2):195–204. 10.1348/135910708X295604 18331667

[31] Hanna KM, Decker CL. A concept analysis: Assuming responsibility for self-care among adolescents with type 1 diabetes. J Spec Pediatr Nurs. 2010;15(2):99–110. 10.1111/j.1744-6155.2009.00218.x 20367781 PMC2851236

[32] Scholes C, Mandleco B, Roper S, Dearing K, Dyches T, Freeborn D. A qualitative study of young people’s perspectives of living with type 1 diabetes: Do perceptions vary by levels of metabolic control?. J Adv Nurs. 2013;69(6):1235–47. 10.1111/j.1365-2648.2012.06111.x 22861071

